# Cold stress induces differential gene expression of retained homeologs in *Camelina sativa* cv Suneson

**DOI:** 10.3389/fpls.2023.1271625

**Published:** 2023-11-16

**Authors:** Chao Fang, John P. Hamilton, Brieanne Vaillancourt, Yi-Wen Wang, Joshua C. Wood, Natalie C. Deans, Taylor Scroggs, Lemor Carlton, Kathrine Mailloux, David S. Douches, Satya Swathi Nadakuduti, Jiming Jiang, C. Robin Buell

**Affiliations:** ^1^ Department of Plant Biology, Michigan State University, East Lansing, MI, United States; ^2^ Center for Applied Genetic Technologies, University of Georgia, Athens, GA, United States; ^3^ Department of Crop & Soil Sciences, University of Georgia, Athens, GA, United States; ^4^ Department of Genetics, University of Georgia, Athens, GA, United States; ^5^ Department of Plant, Soil & Microbial Sciences, Michigan State University, East Lansing, MI, United States; ^6^ Department of Environmental Horticulture, University of Florida, Gainesville, FL, United States; ^7^ Plant Molecular and Cellular Biology Program, University of Florida, Gainesville, FL, United States; ^8^ Department of Horticulture, Michigan State University, East Lansing, MI, United States; ^9^ Institute of Plant Breeding, Genetics & Genomics, University of Georgia, Athens, GA, United States

**Keywords:** camelina, cold stress, genome assembly, homeolog, lipid

## Abstract

*Camelina sativa* (L.) Crantz, a member of the Brassicaceae, has potential as a biofuel feedstock which is attributable to the production of fatty acids in its seeds, its fast growth cycle, and low input requirements. While a genome assembly is available for camelina, it was generated from short sequence reads and is thus highly fragmented in nature. Using long read sequences, we generated a chromosome-scale, highly contiguous genome assembly (644,491,969 bp) for the spring biotype cultivar ‘Suneson’ with an N50 contig length of 12,031,512 bp and a scaffold N50 length of 32,184,682 bp. Annotation of protein-coding genes revealed 91,877 genes that encode 133,355 gene models. We identified a total of 4,467 genes that were significantly up-regulated under cold stress which were enriched in gene ontology terms associated with “response to cold” and “response to abiotic stress”. Coexpression analyses revealed multiple coexpression modules that were enriched in genes differentially expressed following cold stress that had putative functions involved in stress adaptation, specifically within the plastid. With access to a highly contiguous genome assembly, comparative analyses with *Arabidopsis thaliana* revealed 23,625 *A. thaliana* genes syntenic with 45,453 Suneson genes. Of these, 24,960 Suneson genes were syntenic to 8,320 *A. thaliana* genes reflecting a 3 camelina homeolog to 1 Arabidopsis gene relationship and retention of all three homeologs. Some of the retained triplicated homeologs showed conserved gene expression patterns under control and cold-stressed conditions whereas other triplicated homeologs displayed diverged expression patterns revealing sub- and neo-functionalization of the homeologs at the transcription level. Access to the chromosome-scale assembly of Suneson will enable both basic and applied research efforts in the improvement of camelina as a sustainable biofuel feedstock.

## 1 Introduction

*Camelina sativa* (L.) Crantz, also known as false flax or gold-of-pleasure, is a low-cost renewable crop with multiple uses in food, feed, and bio-based applications. It has a broad environmental adaptability with a short life cycle of 85 to 100 days and can be grown in marginalized conditions with minimal agricultural inputs (Vollmann and Eynck, 2015; Malik et al., 2018; Zanetti et al., 2021). Camelina is a member of the Brassicaceae family and produces seeds with up to 40% oil by weight (Rodríguez-Rodríguez et al., 2013; Berti et al., 2016). If blended with conventional jet A fuel in equal proportions, camelina-based biofuel has been shown to reduce particle number and mass emissions by 50–70% (Moore et al., 2017). Key to its use as a sustainable biofuel is the development of camelina cultivars that are adapted to different climates and have favorable seed oil yield and fatty acid profiles. At the biochemical level, the deep knowledge of lipid metabolism in *Arabidopsis thaliana* (hereafter Arabidopsis) has been leveraged to camelina resulting in editing of fatty acid desaturase genes to alter the fatty acid profile in seed oil (Jiang et al., 2017; Morineau et al., 2017; Lee et al., 2021). However, in addition to serving as a storage molecule in seeds, fatty acids are integral components of membranes in which the composition of fatty acids (saturated vs unsaturated) impacts membrane fluidity.

To date, field studies on the impact of climate on camelina have shown that temperature, moisture, and soil type can impact seed yield and fatty acid profiles (Obour et al., 2017; Raziei et al., 2018). Furthermore, a controlled growth chamber experiment revealed that altered temperature resulted in significant changes in seed oil fatty acid profiles (Brock et al., 2020). In acclimation experiments in which spring and winter biotypes were exposed to low and then freezing temperatures, both physiological and gene expression changes were apparent between the biotypes reflecting differential responses to temperature (Anderson et al., 2022; Soorni et al., 2022). Gene expression differences were also observed between a spring and winter biotype following an 8-week cold acclimation period (Wang et al., 2022). In a limited study of genes involved in lipid metabolism, a cold stress treatment (4°C) induced expression of *CsPDAT1-A* and *CsPDAT1-C* that encode phospholipid:diacylglycerol acyltransferases which catalyze the final acylation step in triacylglycerol (TAG) biosynthesis (Yuan et al., 2017). Obtaining a better understanding of the impacts of climate on not only fatty acid and lipid profiles across organs but also other key agronomic traits is critical to developing camelina as a biofuel crop with resilience to climate variation.

The genome sequence of the doubled haploid DH55 *C. sativa* accession (641 Mb assembly) was published in 2014 and encodes ~89,000 genes (Kagale et al., 2014). Comparative genome analyses are consistent with a recent whole genome triplication in camelina that resulted in a highly undifferentiated hexaploid genome structure (Kagale et al., 2014). This is supported by recent chromosome painting, genome *in situ* hybridization, and phylogenetic analyses, which suggests that *C. sativa* is derived from an auto-allotetraploid *C. neglecta*‐like species and the diploid species *C. hispida* (Mandáková et al., 2019). As a polyploid, genome fractionation has occurred in camelina along with subgenome dominance (Kagale et al., 2014). Conserved as well as sub- and neo-functionalization of gene expression among the homeologs has been reported (Kagale et al., 2014; Heydarian et al., 2018; Gomez-Cano et al., 2022). In addition to genomic and a suite of transcriptomic resources (Kagale et al., 2014; Abdullah et al., 2016; Abdullah et al., 2018; Gomez-Cano et al., 2020; Gomez-Cano et al., 2022), population genetics studies have been performed that associate agronomic traits with genomic loci (King et al., 2019; Luo et al., 2019; Chaudhary et al., 2020; Li et al., 2021).

While the DH55 reference genome has been highly useful to the community and a new version (v2) of the reference genome has been released (http://cruciferseq.ca), both genome assemblies are highly fragmented due to exclusive use of short read sequences in the assembly process. The use of long read sequencing platforms, coupled with significantly improved algorithms for genome assembly, permit the construction of a chromosome-scale, high quality camelina genome assembly that can enable an improved understanding gene function including regulation of homeologs. In this study, we generated a chromosome-scale, high quality reference genome sequence and annotation for the spring biotype cultivar ‘Suneson’ which has been widely used by the research community (Na et al., 2018; Ozseyhan et al., 2018; King et al., 2019; Na et al., 2019; Lhamo et al., 2020; Gomez-Cano et al., 2022; Bengtsson et al., 2023). To further our understanding of the impact of gene regulation, we examined gene expression in camelina leaves exposed to cold stress revealing conserved as well as differential gene expression among retained homeologs. The Suneson genome resource will be of value to researchers interested in engineering camelina as a biofuel crop and in understanding genome evolution in polyploids.

## 2 Materials and methods

### 2.1 Plant material

For generation of high molecular weight DNA, *Camelina sativa* cv Suneson seeds were subjected to one round of single seed descent, planted in soil, and grown at 25.5°C day/18°C night for 19 days under a 15 hr photoperiod with 500 μE·m^−2^·s^−1^ of light. Plants were stored in the dark for 24 hours, leaves were harvested, and flash frozen in liquid nitrogen. For the Illumina whole genome shotgun (WGS) library, immature leaves were harvested from plants grown for 22 days in soil at 22°C day/18°C night under 400 μE·m^−2^·s^−1^ of light with a 15 hr photoperiod, and then dark treated for 24 hours prior to DNA isolation. For generation of transcript data to support genome annotation, we harvested mature leaf, immature seed stage 1, immature seed stage 2, stem, open flower, and root without the dark treatment and flash froze tissues in liquid nitrogen prior to RNA isolation.

To examine the response of Suneson to cold treatment, bulk seeds were sterilized three times with 75% ethyl alcohol for 5 minutes and then plated on Murashige and Skoog plates for 4 days prior to transfer to soil. Potted seedlings were grown in a growth chamber at 22°C day/18°C night under a 16 hr photoperiod. After three weeks, plants were exposed to cold temperature (10°C/day, 6°C/night) for 48 hrs; plants not exposed to cold stress were used as a control.

### 2.2 Nucleic acid isolation, library construction, and sequencing

High molecular weight genomic DNA was isolated from dark-treated immature leaves using the Takara Bio Nucleobond HMW DNA Kit (Takara Bio USA, San Jose CA); short fragments were eliminated using the Short Read Eliminator kit (Pacific Biosciences, Menlo Park, CA). Oxford Nanopore Technologies (ONT) libraries were constructed using the Ligation Sequencing Kit (SQK-LSK114, Q20+ chemistry) and sequenced on FLO-MIN114 flow cells as described previously (Li et al., 2023b) (**Supplementary Table 1**). Bases were called using Guppy v6.3.7 in super high accuracy mode (https://nanoporetech.com/community). DNA for error correction was isolated from dark-treated immature leaves using the Qiagen genomic tip method (Vaillancourt and Buell, 2019) and WGS libraries were constructed using the PerkinElmer NEXTFLEX Rapid XP DNA-Seq Kit HT (Perkin Elmer, Waltham, MA) (**Supplementary Table 2**). Libraries (five in total) were sequenced on an Illumina NovaSeq 6000 in paired-end mode generating 150 nt reads.

### 2.3 Genome assembly and chromosome scaffolding

Jellyfish (v2.2.10) (Marçais and Kingsford, 2011) was used to count k-mers (k = 21) in the WGS reads which were analyzed with GenomeScope (v2.0) (Vurture et al., 2017) to determine the extent of heterozygosity and shared k-mers among the homeologs. In addition, a smudgeplot was generated using Smudgeplot (v0.2.5) (Ranallo-Benavidez et al., 2020) with k-mers (k = 21) counted with KMC (v3.1.1) (Kokot et al., 2017). ONT gDNA reads were filtered using seqtk (v1.3) (https://github.com/lh3/seqtk) to remove reads less than 15 kb. The genome was assembled using Flye (v2.9.1) (Kolmogorov et al., 2019) with the genome size set to .785g, zero polishing iterations, and asm-coverage 60. The initial assembly was error-corrected through two rounds of Medaka (v1.7.2; https://github.com/nanoporetech/medaka) with all of the ONT genomic DNA reads using the model r1041_e82_400bps_sup_g615. This was followed by two rounds of Pilon (v1.24) (Walker et al., 2014) using the alignments from Cudadapt-trimmed reads (v4.1) (Martin, 2011) that were aligned to the assembly using bwa-mem2 (v2.2.1) (Li, 2013). Contigs less than 50 kb were filtered out using seqkit (v2.3.0) (Shen et al., 2016). Two rounds of RagTag (v2.1.0) (Alonge et al., 2022) was used to generate a chromosome-scale assembly using the reference genome DH55 v2.0 (http://cruciferseq.ca). Kraken 2 (v2.1.2) (Wood et al., 2019) was used to check all reads and the final assembly for contamination. KAT (v 2.4.1) (Mapleson et al., 2017) was used to determine the representation of k-mers in the final assembly. Genome completeness was assessed using Benchmarking Universal Single Copy Orthologs (BUSCO, v5.4.3) (Waterhouse et al., 2018) with the embryophyta_odb10 database.

### 2.4 Preparation of RNA-seq and full-length cDNA libraries

To support high quality gene annotation, RNA was isolated from a diverse set of tissues using either the hot borate method (Wan and Wilkins, 1994) (mature leaf and stem) or Purelink RNA isolation kit (Thermo Fisher Scientific, Waltham MA) (immature seed stage 1, immature seed stage 2, open flower, and root). Total RNA was treated with Turbo DNAse (Thermo Fisher Scientific, Waltham MA) following the manufacturer’s directions. ONT cDNA libraries were constructed using the SQK-PCB109 library preparation kit (Oxford Nanopore Technologies, Oxford UK) and sequenced on FLO-MIN106 flow cells (**Supplementary Table 1**). Bases were called using Guppy v6.3.7 (https://nanoporetech.com/community) in the super high accuracy mode with barcode trimming disabled.

For cold stress experiments, two biological replicates of leaf tissue were collected from control and cold-treated plants and ground into a fine powder in liquid nitrogen. Total RNA was extracted using the RNeasy Plant Mini Kit (Qiagen, Germantown MD) and RNA-seq libraries were constructed using the KAPA mRNA HyperPrep Kit protocol (KAPA Biosystems, Wilmington, MA). RNA-seq libraries were sequenced in paired-end mode generating 150 nt reads on an Illumina NovaSeq 6000 (**Supplementary Table 1**). ONT cDNA libraries were constructed, sequenced, and bases called as described above.

### 2.5 Genome annotation

The Suneson genome was annotated for protein-coding genes as described previously (Pham et al., 2020). In brief, repetitive sequences were identified using RepeatModeler (v2.0.3) (Flynn et al., 2020) from which protein-coding genes were removed using Protex (v1.2)(Campbell et al., 2014). These filtered repeat sequences were added to the Repbase Viriplantae repeat dataset (v20150807) to construct a final repeat library. Prior to annotation, RepeatMasker (v4.1.2-p1) (Chen, 2004) was used to mask the genome using the parameters -s -nolow -no_is -gff. RNA-seq reads were cleaned of low quality sequences and adapters using Cutadapt (v2.10) (Martin, 2011) with a quality cutoff of 10 and a minimum length of 100nt (**Supplementary Table 2**). Cleaned reads were aligned to the Suneson genome using HISAT2 (v2.1.0) (Kim et al., 2019) with a maximum intron length of 5000 and genome-guided transcript assemblies were generated using Stringtie 2 (v2.2.1) (Kovaka et al., 2019). The BRAKER2 pipeline (v2.1.6) (Hoff et al., 2019) was used to predict gene models using the RNA-Seq alignments as hints. Gene models were refined through two rounds of PASA (v2.5.2) (Haas et al., 2003; Campbell et al., 2006) using the RNA-seq and ONT cDNA reads resulting in a set of 145,971 working gene models. To identify high confidence gene models, gene expression data were generated using Kallisto (v0.46.2) (Bray et al., 2016) with the mRNAseq reads and Stringtie (v2.2.1) (Kovaka et al., 2019) with the ONT cDNA reads. Predicted proteins were searched against the Arabidopsis v11 predicted proteome (Araport.org) using Diamond (v0.9.36) (Buchfink et al., 2015) and Pfam domains were identified using the PFAM database (v32.0) (El-Gebali et al., 2019) with HMMER (v3.3) (Mistry et al., 2013). High confidence gene models were determined based on gene expression (TPM > 0) and/or protein match to Arabidopsis and/or presence of a Pfam domain. Functional annotation was assigned to the gene models using matches to Arabidopsis, the presence of Pfam domains, and expression evidence. Transcription factors were predicted using iTAK v1.7 (Zheng et al., 2016) with the high-confidence representative peptide sequences.

### 2.6 Gene expression abundances, differential gene expression, and gene coexpression analyses

Gene expression abundance estimations were calculated for cold-stressed and control leaves (this study) along with publicly available data that was downloaded from the National Center for Biotechnology Information Sequence Read Archive. First, reads were cleaned using Cutadapt (v4.1) (Martin, 2011) with a minimum read length of 40, 3’ end quality cutoff of 30, flanking N base removal, and 3’ adapter sequence trimming. The Kallisto quant algorithm (v0.48.0) (Bray et al., 2016) was used to quantify expression with a k-mer size of 21; libraries that were sequenced in single end mode were run with two additional parameters, a fragment length of 200 and standard deviation of 20. Libraries that were sequenced in paired end mode were run with the –rf-stranded parameter. Gene coexpression networks were constructed using Simple Tidy GeneCoEx in R (Li et al., 2023a) with genes that had a TPM > 1.

To detect differential gene expression, RNA-seq reads were mapped to Suneson genome using HISAT2 (version 2.0.0-beta) (Kim et al., 2019) and expression abundances were calculated by StringTie (v1.3.3b) (Pertea et al., 2016) to determine gene expression abundances. Significantly differentially expressed homeologous genes among a triplet (*p* < 0.01) or between cold and control samples (|log2FC| > 1; *p* < 0.01) were identified using EdgeR (Robinson et al., 2010).

### 2.7 Homeologous gene identification

Genome annotation for *Arabidopsis lyrata* and *A. thaliana* (Araport11) was downloaded from Phytozome (v13) (Goodstein et al., 2012). The GENESPACE pipeline (Lovell et al., 2022) was run with the Suneson genome and *A. thaliana* using GENESPACE v0.9.3 to identify triplicated homeologs within Suneson relative to *A. thaliana*. For *A. lyrata*, GENESPACE v1.1.4 was used with the representative gene model annotations to identify syntelogs between Suneson and *A. lyrata*. The default pipeline options were used except for the ploidy option which was set to 1,3. The syntelogs were exported from the pan-genome databases using the query_pangenes GENESPACE function.

### 2.8 Gene expression of triplicated homeologous genes

We first classified variation in expression across triplicated homeologs under control conditions by ranking the three homeologous genes based on their average FPKM value. If the highest expressed gene in a triplet of homeologs showed a significantly higher expression level (*p < *0.01 and fold change of FPKM > 2) than the other two copies, this homeolog was classified as a Class 1 homeolog. If two genes in the triplet showed a significantly higher expression level (*p < *0.01 and fold change of FPKM > 2) than the third copy, this homeolog was classified as a Class 2 homeolog. If all of the copies of a triplet showed similar expression levels (fold change of FPKM between every two copies < 1.5), this homeolog was classified as a Class 3 homeolog. Gene ontology analyses of the three classes of homeologs were performed and displayed using TBtools (Chen et al., 2020).

In the response to cold stress, if a triplicated homeolog had a significantly higher expression level following cold stress relative to the control sample, this gene was termed a cold-induced gene. A cold-induced homeolog was classified as a Type 1 triplet if all three homeologs were cold-induced; a homeolog was classified as a Type 2 homeolog if two of the three copies were cold-induced; and a homeolog was classified as a Type 3 homeolog if one of the three copies was cold-induced. For every homeolog in a Type 1 triplet, we calculated the fold change of its FPKM value between cold-treated and control samples and used the fold change to represent the cold response level of this homeolog. We ranked the three homeologous genes based on their cold inducibility with the copy exhibiting the highest level ranked first and the copy with the lowest level ranked third. The cold inducibility of the first and third copies were compared to detect the divergence of their response to cold stress.

## 3 Results and discussion

### 3.1 Genome assembly of *C. sativa* cv Suneson

As the camelina genome is a hexaploid, we assessed the number of unique k-mers in the Suneson genome using Illumina WGS reads. The k-mer distribution plot (**Supplementary Figure 1**) is consistent with a diploidized genome in which the majority of k-mers (k = 21) were present in single copy with a subset present in two copies and an even smaller subset in three copies. We also examined the pattern of near-identical k-mers using SmudgePlot (**Supplementary Figure 2**) in which 49% of the k-mer pairs were present as AAB, 46% present as AB, and 5% as AAAB, consistent with the hypothesized origin of hexaploid camelina being derived from an auto-allotetraploid *C. neglecta*‐like species and the diploid species *C. hispida* (Mandáková et al., 2019). Using ~42× coverage ONT genomic reads greater than 15 kb and the Flye assembler software, we assembled 647,473,868 bp of the Suneson genome into 551 contigs with an N50 contig length of 12,024,690 bp (**Supplementary Table 3**). Two rounds of error correction with Medaka followed by two rounds of Pilon were performed. The assembly was filtered to remove contigs less than 50 kb, yielding a 644,482,469 bp assembly contained in 157 contigs with an N50 length of 12,031,512 bp (**Supplementary Table 3**). To assemble to the 20 camelina chromosomes, Ragtag was used with the DH55 reference assembly resulting in 98.3% of the Suneson assembly anchored to the chromosomes (**Supplementary Table 4**). To validate the assembly, we used the KAT program to determine the representation of WGS-derived k-mers in the final assembly. As shown in **Supplementary Figure 3**, the majority of k-mers were present in single copy within the assembly with limited numbers present at two copies and a small set of k-mers present in three copies. To assess the representation of genic sequences in the genome assembly, we ran BUSCO with the embryophyta_odb10 database. A total of 1,606 of the 1,614 (99.5%) BUSCO orthologs were complete in the Suneson assembly, of which, 1,581 (98.0%) are duplicated as expected due to the hexaploid nature of the camelina genome; a mere 0.2% and 0.3% were fragmented or missing, respectively (**Supplementary Table 5**).

### 3.2 Genome annotation

Repetitive sequences in the Suneson genome were identified using a combination of *de novo* repeat identification and sequence similarity to existing Viridiplantae repetitive sequences. In total, 44.2% of the genome was annotated as repetitive (**Supplementary Table 6**), which is substantially higher than the percentage (25%) identified in the DH55 assembly which is attributable to the short-read-derived DH55 genome sequence. Retroelements (25%) dominated the annotated repetitive sequences relative to DNA transposons (2.85%). Annotation of protein-coding genes using five mRNA-seq libraries and full-length cDNA sequences derived from eight different tissues (**Supplementary Table 7**) resulted in 145,971 working gene models from 103,435 loci (**Supplementary Table 8**). Of these working models, 133,355 were high confidence models derived from 91,877 loci. While the number of total genes was similar between the Suneson and DH55 v2 annotation, access to substantial full-length cDNA sequence data permitted annotation of more gene models in the Suneson assembly compared to the DH55 assembly. In the Suneson annotation, the average number of gene models per locus in the working and high confidence gene sets to 1.41 and 1.45, respectively, which is higher compared to the average of 1.06 gene models per locus in the DH55 annotation (**Supplementary Table 8**). To assess the quality of the genome annotation, we examined the representation of BUSCO orthologs. Within the representative high confidence gene model set, 98.4% of the BUSCO orthologs were present with 92.8% present as duplicated, consistent with the hexaploid nature of the camelina genome (**Supplementary Table 5**).

**Figure 1:**
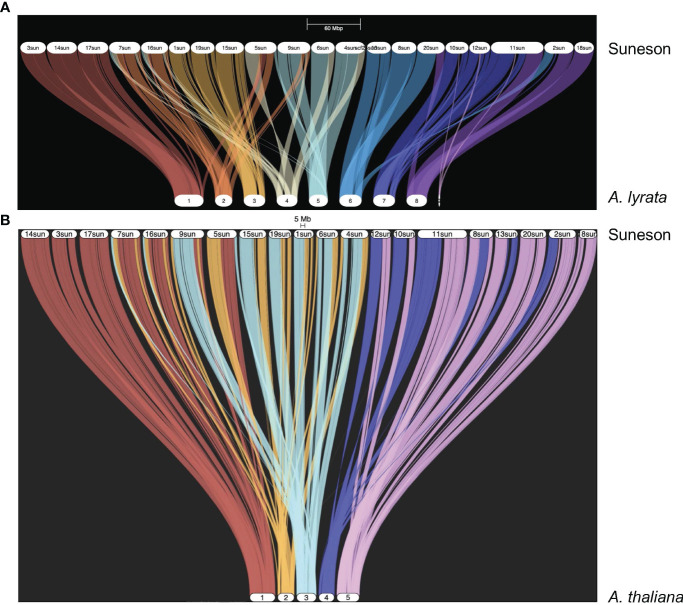
Syntelogs between *Arabidopsis* species and *Camelina sativa* cv Suneson. GENESPACE was used to identify syntelogs **(A)** between *Arabidopsis lyrata* and *Camelina sativa* cv Suneson and **(B)** between *Arabidopsis thaliana* and *Camelina sativa* cv Suneson.

Kagale et al. (2014) reported a high degree of synteny between *C. sativa* and *A. lyrata.* In the Suneson genome, 59,645 genes were syntenic to 20,934 *A. lyrata* genes as shown in the riparian plot of synteny between these two species (**Figure 1A**). To understand the relationship of the subgenomes within Suneson, we identified syntelogs between *A. thaliana* and Suneson using GENESPACE resulting in 23,625 *A. thaliana* genes syntenic with 45,453 Suneson genes (**Supplementary Table 9**). The riparian plot of the three subgenomes relative to the five chromosomes of *A. thaliana* (**Figure 1B**) highlights the significant degree of conservation between *A. thaliana* and the three subgenomes in Suneson. Of these, 24,960 Suneson genes were syntenic to 8,320 *A. thaliana* genes reflecting a 3 camelina homeolog to 1 A*. thaliana* relationship and retention of all three homeologs (**Supplementary Table 10**). In addition to fully retained homeologs, 2,829 *A. thaliana* genes were syntenic to 5,658 *C. sativa* genes (1:2 ratio) while 7,253 *A. thaliana* genes were syntenic to 7,253 *C. sativa* genes (1:1 ratio).

### 3.3 Response of camelina leaves to cold stress

**Figure 2:**
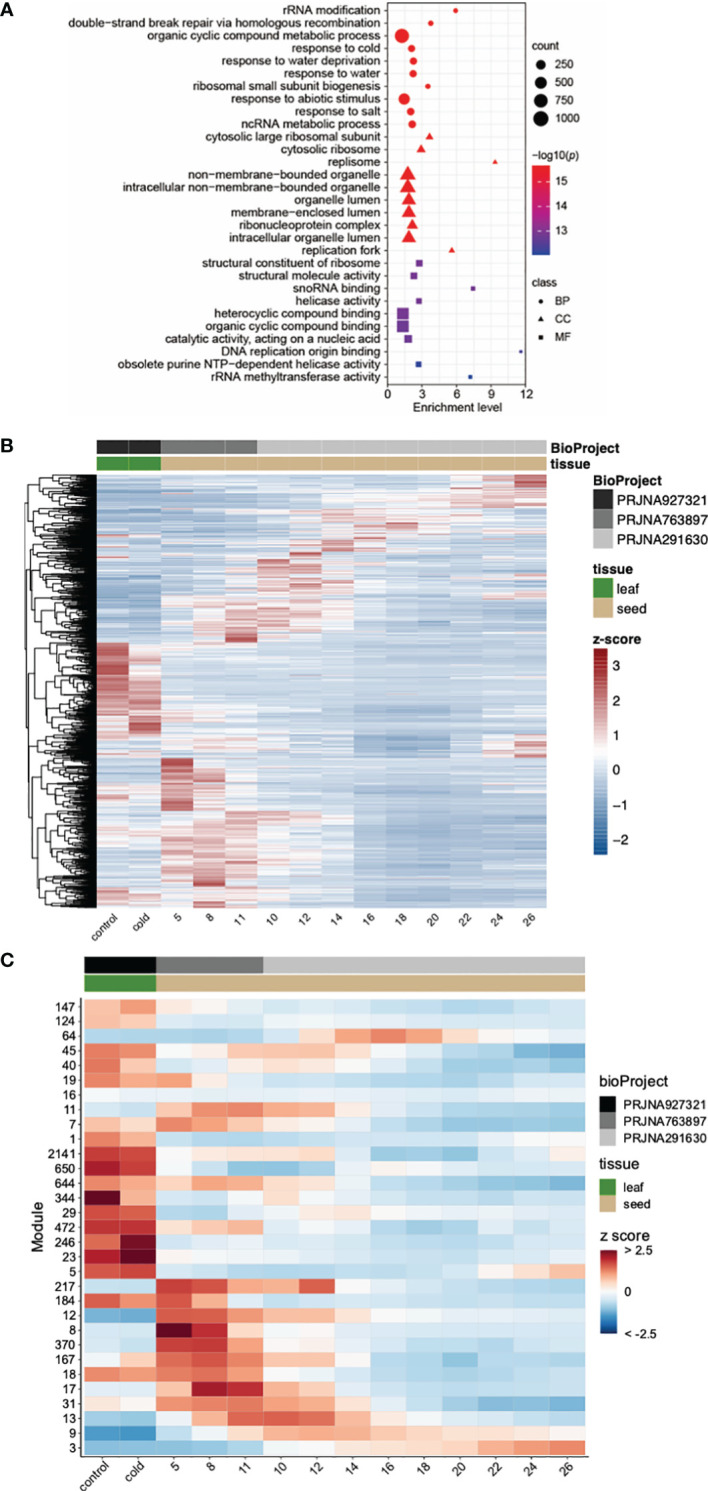
Gene expression in *Camelina sativa* cv Suneson following cold stress. **(A)** Gene ontology (GO) terms enriched in cold induced genes. Only the top 10 enriched GO terms are shown here. A complete list of enriched GO terms is included in **Supplementary Table 13**. Sizes of symbols reflect numbers of genes, color reflects log_10_ *p*-value and symbols reflect GO categories (BP: Biological process; CC: Cellular compartment; MF: Molecular function). **(B)** Gene expression in *C. sativa* cv Suneson leaf and developing seeds. Gene expression abundances for 1,452 genes involved in fatty acid and lipid metabolism were calculated using Kallisto for control and cold stressed leaves (this study) and two sets of seed development series obtained from the National Center for Biotechnology Information Sequence Read Archive. Numbers for seed samples reflect days post anthesis. **(C)** Modules of coexpressed genes using gene expression abundances from control and cold-stressed leaves (this study) and two seed development studies. Numbers for seed samples reflect days post anthesis.

We performed RNA-seq using leaf tissue collected from cold-treated plants to gain insight on the impact of short-term cold stress on leaf tissue. A total of 4,467 genes showed a significantly higher expression level in cold-treated samples compared to their expression under control temperature; 4,851 were down-regulated under cold stress (**Supplementary Tables 11**, **12**). Gene ontology terms related to stress response were enriched in cold-inducible genes including “response to cold”, “response to temperature stimulus”, and “cold acclimation” (**Figure 2A**; **Supplementary Table 13**).

As temperature impacts fatty acid and lipid composition, we examined the expression of genes involved in lipid and fatty acid metabolism under cold stress in Suneson leaves. Using 552 A*. thaliana* genes previously associated with lipid and fatty acid metabolism (Nguyen et al., 2013), we identified 1,474 genes in the Suneson assembly involved in lipid and fatty acid metabolism (**Supplementary Table 14**). Expression of these genes were strikingly different between leaf and developing seeds as shown in **Figure 2B** consistent with the diverged function of lipid metabolism in these tissues. Of these, 60 were differentially up-regulated in cold stressed leaves while 111 were down-regulated. Multiple genes involved in remodeling membrane lipids were upregulated (**Supplementary Table 11**) including lipid transfer proteins functioning in phospholipid transfer between cell membranes. Also up-regulated were genes encoding phosphoinositide-specific phospholipase C which is associated with hormone signaling, abiotic stresses, and pathogen responses (Rupwate and Rajasekharan, 2012). Notably, a *DIACYLGLYCEROL KINASE (DGK)* gene was upregulated when exposed to cold temperatures (**Supplementary Table 11**). As plants balance the levels of phosphatidic acid, diacylglycerol, and triacylglycerol during cold stress, DGKs plays a major role in remodeling cold-responsive lipids (Tan et al., 2018). We did not observe up-regulation of phospholipid:diacylglycerol acyltransferases which have been reported to be induced by cold stress (Yuan et al., 2017) and shown to enhance fitness under cold stress in *A. thaliana* (Demski et al., 2020). This may be attributable to the warmer and shorter cold stress conditions employed in this study which may not have been sufficient to induce gene expression. In contrast, different classes of lipoxygenases (LOX gene family) involved in lipid catabolism and the formation of oxylipins including the defense-related hormone jasmonic acid were downregulated under cold stress (**Supplementary Table 12**) consistent with a previous study (Zhu et al., 2018). Oxylipins have been reported to play a role in cold stress through jasmonic acid-mediated regulation of Inducer of CBF (ICE) – C-Repeat Binding Factor (CBF)/DRE Binding Factor 1 through the alleviation of oxidative damage in cells (Hu et al., 2013).

To identify genes that are co-regulated under cold stress, we constructed gene coexpression networks. After filtering for the top 20% variable genes and an r > 0.7, 14,765 genes with 13,803,988 edges were used to construct coexpression modules (**Figure 2C**; **Supplementary Table 15**). With respect to gene expression following cold stress, four modules were of interest (Modules 5, 23, 124, 167). Module 5 contained 233 genes, of which, 62 were up-regulated and 22 were down-regulated following cold stress. Genes in Module 5 were enriched in GO terms associated with response to light, abiotic stress, and environmental stimuli as well as regulation of genes involved in photosynthesis (**Supplementary Table 16**). With respect to cellular compartment, Module 5 genes were associated with the plastid and the peroxisome. Module 23 (93 genes) had 35 and 4 genes up-regulated and down-regulated, respectively, in response to cold stress; GO terms associated with Module 23 were associated with response to abiotic stress, cold, and temperature stimulus and were associated with plastic cellular compartment (**Supplementary Table 16**). Similar to Module 5, Module 124 included 99 genes, of which, 24 were up-regulated and 29 were down-regulated and associated with GO terms involved in response to light and abiotic stimuli with localization within the plastid compartment (**Supplementary Table 16**). While only containing 38 genes, Module 167 had 23 genes up-regulated and a single gene down-regulated following cold-stress; GO associations suggest this module was associated with DNA repair (**Supplementary Table 16**).

**Figure 3:**
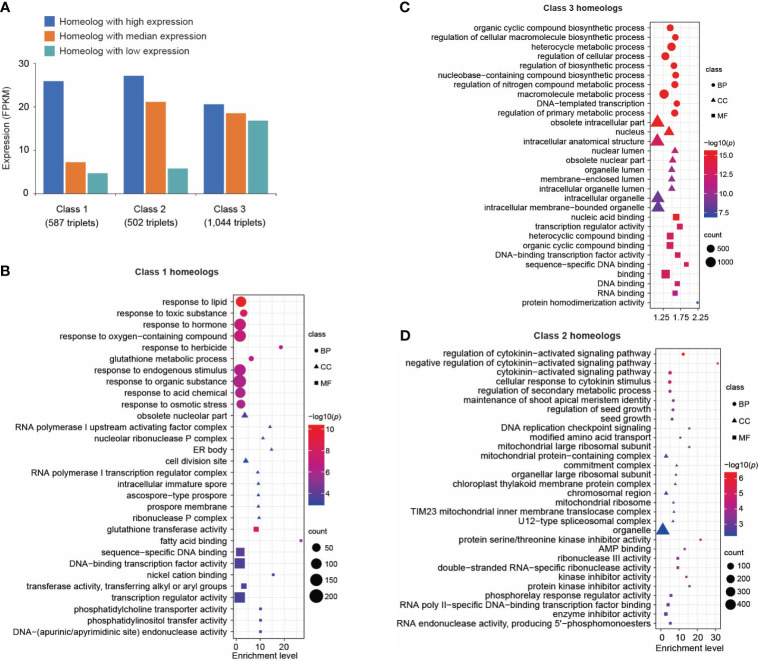
Transcriptional divergence of triplicated homeologous genes in control leaves. **(A)** Classification of three classes of triplicated homeologous genes based on gene expression. Class 1 represents the homeolog in which the highest expressed gene showed a significantly higher expression level (p < 0.01 and fold change of FPKM > 2) than the other two copies. Class 2 represents the homeolog in which two genes showed a significantly higher expression level (*p < *0.01 and fold change of FPKM > 2) than the third copy. Class 3 represents the homeologs in which three copies showed similar expression levels (fold change of FPKM between every two copies < 1.5). **(B)** Gene ontology (GO) terms enriched in Class 1 genes. Only the top 10 enriched GO terms are shown. A complete list of enriched GO terms is included in **Supplementary Table 17**. **(C)** GO terms enriched in Class 3 genes. Only the top 10 enriched GO terms are shown here. A complete list of enriched GO terms is included in **Supplementary Table 18**. **(D)** GO terms enriched in Class 2 genes. Only the top 10 enriched GO terms are shown here. A complete list of enriched GO terms is included in **Supplementary Table 19**. Sizes of symbols reflect numbers of genes, color reflects log_10_ *p*-value and symbols reflect GO categories (BP, Biological process; CC, Cellular compartment; MF, Molecular function).

### 3.4 Expression patterns of retained homeologous genes in *Camelina sativa*

We analyzed the transcriptional divergence of triplicated homeologous genes in the Suneson genome. We identified 8,320 sets of triplicated homeologous genes (see Methods) and analyzed their expression in leaf tissue. We first investigated the expression patterns of these homeologs under control conditions. Among these homeologs, over 76% (6,323/8,320) exhibited expression of all three homeologous genes, while 6.0% (499/8,320) and 6.4% (529/8,320) of the triplets displayed expression of only one and two of the three homeologous copies, respectively. All three copies of the remaining 969 (11.6%) triplets were not expressed in control leaf tissue.

The triplicated homeologs were then cataloged into Class 1, 2, and 3 based on the expression levels of the three homeologs. We identified 587 triplets (Class 1) in which the expression level of one copy is significantly higher (*p* < 0.01) than both of the two other copies (**Figure 3A**). We performed GO analysis on this group of genes. Interestingly, genes responsive to stimuli such as chemicals, stress, and endogenous stimuli were highly enriched in this group (**Figure 3B**; **Supplementary Table 17**). These data suggest neofunctionalization at the expression level among these homeologs. A similar result was reported in *A. thaliana* in which one copy of duplicated genes tends to retain their ancestral stress responses following gene duplication (Zou et al., 2009). We identified 1,044 triplicated homeologs (Class 3) in which the three copies showed a similar level of expression (**Figure 3A**); specifically, the fold change in expression between any two of the three copies was less than 1.5. Gene ontology analysis of this group of genes revealed enrichment of genes related to fundamental processes including biosynthetic processes, metabolic processes, cellular processes, developmental processes, and rhythmic processes (**Figure 3C**; **Supplementary Table 18**). This result indicates that it is favorable to retain expression of all three copies of genes related to fundamental biological processes. For comparison, we identified a set of 502 triplicated homeologs (Class 2) in which the expression levels of two copies were significantly higher (*p* < 0.01) than the third copy (**Figure 3A**); GO analysis revealed that signaling pathways were highly enriched in this class of genes (**Figure 3D**; **Supplementary Table 19**).

In allopolyploids, genes from one subgenome were often preferentially retained or achieved a higher level of expression than those from other subgenomes, which is known as subgenome dominance and has been documented in an increasing number of plant species (Thomas et al., 2006; Schnable et al., 2011; Alger and Edger, 2020). If one of the parental progenitors of an allopolyploid is highly adapted to the environment where the polyploid species originated, then genes responsible for environmental adaptation from this progenitor may be preferentially retained. For example, disease resistance genes were found to be preferentially retained and associated with subgenome dominance in strawberry (Barbey et al., 2019; Edger et al., 2019) and *Brassica napus* (de Jong and Adams, 2023). As noted above, in *A. thaliana* one copy of duplicated genes tends to retain their ancestral stress responses following gene duplication (Zou et al., 2009). Here, we show in *C. sativa* that a specific homeolog of genes responsive to stimuli tends to gain dominance in transcription in comparison to other homeologs. These results suggest that stress responsive genes have a distinct evolutionary trajectory in the evolution of allopolyploid species.

### 3.5 Diverged responses to cold stress among retained homeologous genes

**Figure 4:**
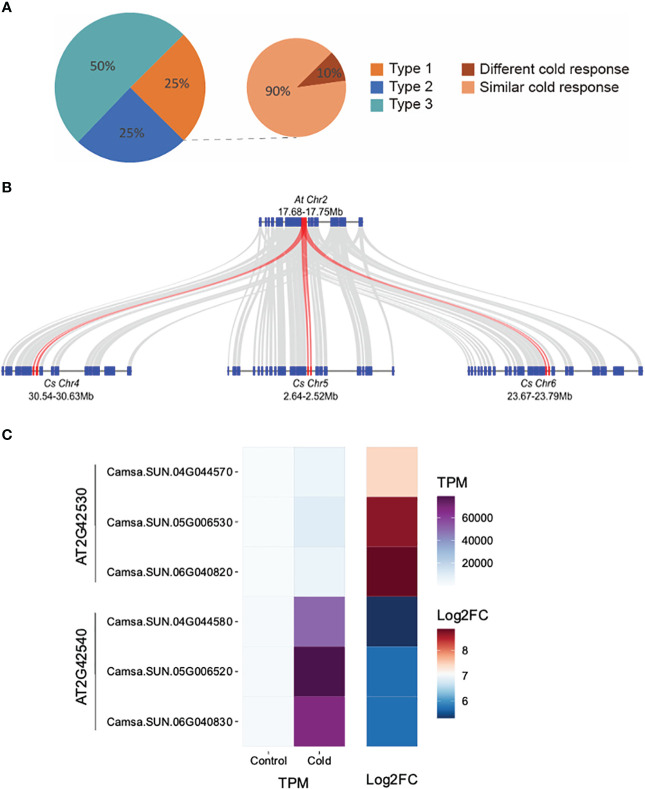
Transcriptional divergence of triplicated homeologous genes in *C. sativa* leaves following cold stress. **(A)** Classification of the three types of cold-response triplicated homeologs. The three homeologous genes of Type 1 triplets were all cold-induced; Two of the three homeologs from Type 2 triplets were cold-induced; Only one of the three homeologs from Type 3 triplets was cold-induced. “Different cold response” indicates the Type 1 triplets in which one homeolog showed at least two-times higher cold inducibility than the two other homeologs. The remaining Type 1 triplets are termed as “similar cold response”. **(B)** McScan was used to display the systemic relationship of *C. sativa* homeologs of *Arabidopsis thaliana COR15* genes (red) and flanking genes. **(C)** Gene expression of *COR15* homeologs in control and cold-treated leaves.

We investigated how the expression of retained triplicated homeologous genes evolved in their response to an environmental cue, that of cold stress. Of the 4,467 genes up-regulated in response to cold stress, 36.5% (1,632/4,467) belong to 935 triplicated homeologs. We classified the 935 homeologs into three types. Type 1: all three homeologous copies were cold-inducible; Type 2: two of the three copies were cold-inducible; Type 3: only one copy was cold inducible. Our analysis revealed that 75% of the triplicated homeologous genes displayed diverged cold responses as at least one homoeologous copy was not induced after cold treatment (Type 2 or 3) (**Figure 4A**). Examples of Type 1 triplicated homeologs with retained expression are Camsa.SUN.04G044570, Camsa.SUN.04G044580, Camsa.SUN.05G006530, Camsa.SUN.05G006520, Camsa.SUN.06G040820, and Camsa.SUN.06G040830 which are syntelogs with the *A. thaliana* cold-regulated (*COR*) genes AT2G42530 (*COR15b*) and AT2G42540 (*COR15a*) present in tandem on *A. thaliana* chromosome 2 (**Figure 4B**). Arabidopsis COR15a and COR15b are small chloroplast-targeted polypeptides induced under cold stress, localized in the chloroplast stroma which function in freezing tolerance (Lin and Thomashow, 1992a; Lin and Thomashow, 1992b; Wilhelm and Thomashow, 1993; Artus et al., 1996; Thomashow, 1998; Thalhammer and Hincha, 2013). While all six Suneson genes are up-regulated in response to cold stress, the triplicated homeologs differ in basal gene expression levels and in the extent of up-regulation (**Figure 4C**). Syntelogs of AT2G42530 had lower basal expression but higher log2 fold-change relative to the AT2G42540 syntelogs which had a higher basal expression but lower log2 fold-change (**Figure 4C**). In addition, the extent of cold-induction within each set of the triplicated homeologs differed. For example, the log2 fold-change of Camsa.SUN.04G044570 is lower than Camsa.SUN.05G006530 and Camsa.SUN.06G040820 (**Figure 4C**). Similar cold-specific expression of the *Wcor15* homeolog has been documented in allopolyploid wheat and suggested to play an important role in cold hardiness in wheat and barley (Takumi et al., 2003).

To further compare the “cold inducibility” of each gene within all of the 233 Type 1 triplicated homeologs, we calculated the fold change in expression levels between control and cold treatment sample. We then ranked the three homeologous copies of each triplicated homeolog based on their cold inducibility, with the copy exhibiting the highest expression level ranked first and the copy with the lowest level ranked third (**Figure 4A**). Our analysis revealed that >10% of the Type 1 homeologs exhibited a two-fold or greater difference in cold inducibility between the first and third-ranked copies, suggesting divergence in the degree of cold inducibility among the homeologous genes. Such homeolog expression bias, where one homeolog is preferentially expressed relative to the other, has been reported in multiple other allopolyploid species including *Gossypium* (Hovav et al., 2008; Flagel and Wendel, 2010), *Triticum* (Bottley et al., 2006; Wei et al., 2019), *Brassica* (Auger et al., 2009; Wu et al., 2018; Lee and Adams, 2020), and other species (Grover et al., 2012). Abiotic stress conditions, especially cold stress, considerably impacts expression bias of homeologs involved in physiological responses. Homeologs with differential gene expression are involved in the CBF-COR signaling pathway, fatty acid metabolism which impacts plasma membrane fluidity and stabilization, scavenging reactive oxygen species, sucrose metabolism, and accumulation of secondary metabolites, all which contribute to cold tolerance (Combes et al., 2013; Lee and Adams, 2020; Park and Jang, 2020; Wu et al., 2022). Therefore, studying homeolog gene expression and their sub-functionalization provides foundational knowledge that can be utilized in engineering cold tolerant camelina.

## 4 Conclusions

Access to chromosome-scale genome assemblies and high quality annotation have been foundational resources for genomics-enabled improvement of crop plants. These data have facilitated the understanding of genetic diversity, population structure, structural variation, and quantitative genetics across many crop species. For camelina to be an adaptable biofuel feedstock, improvements in agronomic performance and optimization of seed oil composition and yield will be required. This will entail both conventional breeding and biotechnological approaches that will be enabled by tapping into genetic diversity (Luo et al., 2019; Li et al., 2021) and facile transformation via floral dip (Liu et al., 2012). Access to a chromosome-scale, highly contiguous genome assembly for the widely used spring biotype Suneson was generated in this study and will enable not only basic research on molecular, physiological, and biochemical traits but also breeding cultivars with improved agronomic and biofuel traits. In addition to generation of a chromosome-scale assembly of Suneson and classification of syntelogs with two *Arabidopsis* species, we documented the transcriptional response to cold stress in vegetative leaves including identification of differentially expressed genes, generation of coexpression modules, and characterization of conserved/diverged expression of homeologous genes. These datasets provide a foundation for more detailed interrogation of gene function and regulation in camelina as well as how these diverged from the model species, *A. thaliana*.

## Data availability statement

The datasets presented in this study can be found in online repositories. The names of the repository/repositories and accession number(s) can be found below: Raw sequence reads for all generated data are available through the National Center for Biotechnology Information Sequence Read Archive under BioProject ID PRJNA927321. The genome assembly, annotation, gene expression abundances and GENESPACE results are available on Figshare (https://figshare.com/s/6f95ce23f7c4eded54d6).

## Author contributions

CF: Formal Analysis, Methodology, Writing – original draft, Writing – review & editing, Data curation, Investigation, Software, Visualization. JH: Data curation, Formal Analysis, Investigation, Methodology, Software, Visualization, Writing – original draft, Writing – review & editing. BV: Data curation, Formal Analysis, Investigation, Methodology, Software, Visualization, Writing – original draft, Writing – review & editing. Y-WW: Data curation, Formal Analysis, Investigation, Methodology, Software, Visualization, Writing – review & editing. JW: Investigation, Methodology, Writing – review & editing, Supervision. ND: Investigation, Writing – review & editing, Formal Analysis, Software, Visualization. TS: Writing – review & editing, Methodology. LC: Methodology, Writing – review & editing. KM: Methodology, Writing – review & editing, Supervision. DD: Supervision, Writing – review & editing, Conceptualization, Funding acquisition. SN: Conceptualization, Funding acquisition, Writing – review & editing, Formal Analysis, Investigation, Resources, Writing – original draft. JJ: Conceptualization, Funding acquisition, Investigation, Writing – review & editing, Supervision. CRB: Conceptualization, Funding acquisition, Supervision, Writing – review & editing, Formal Analysis, Methodology, Project administration, Resources, Writing – original draft.
