# Supplementary material for: Cold stress induces differential gene expression of retained homeologs in Camelina sativa cv Suneson
Source: Front Plant Sci. 2023 Nov 16;14:1271625. doi: 10.3389/fpls.2023.1271625 (PMC10687638; doi:10.3389/fpls.2023.1271625)
Supplement: Supplementary file 1 [file Image_1.pdf]

Supplementary Material

1.1 Supplementary Figures

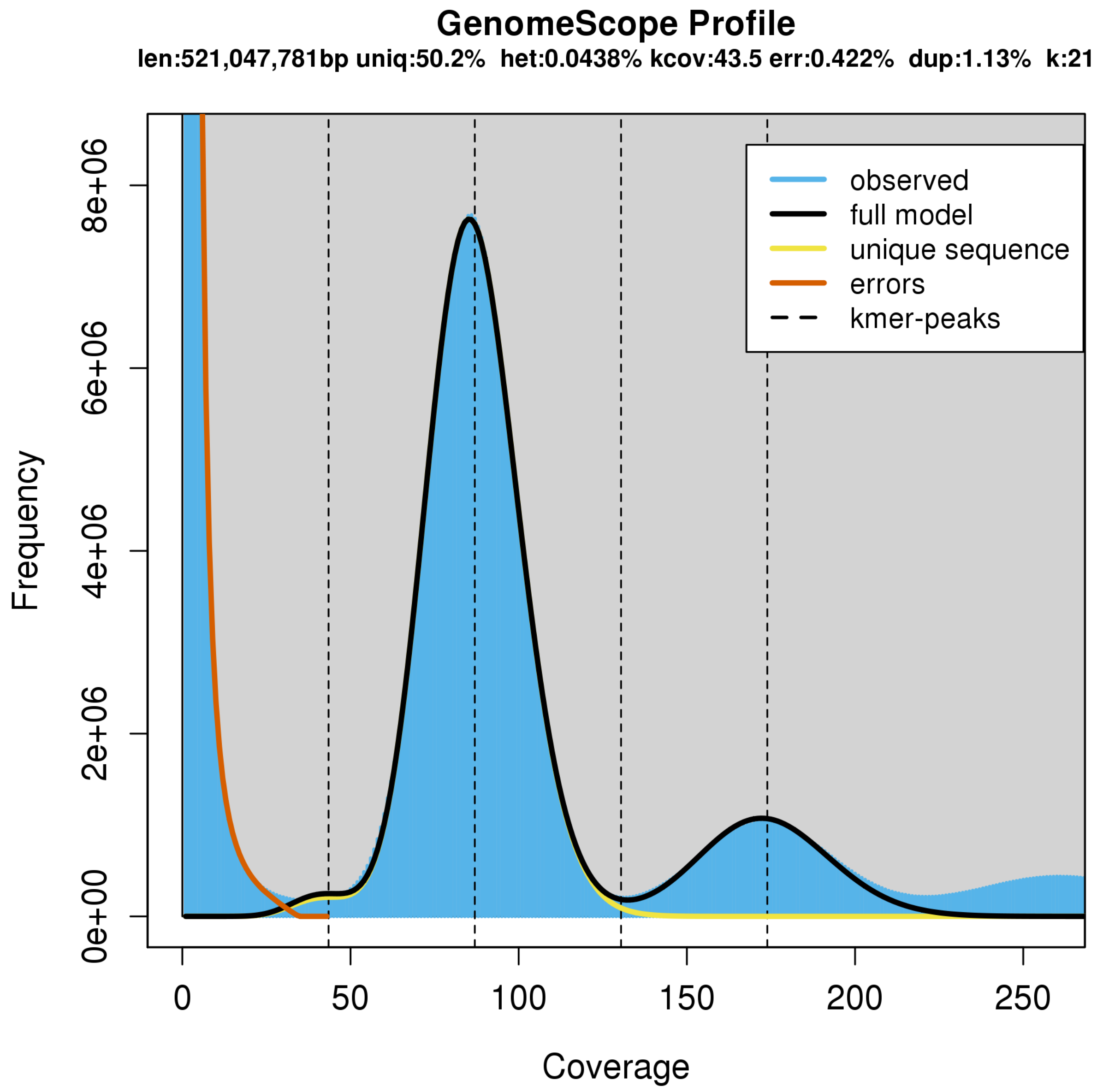

Supplementary Figure 1. GenomeScope k-mer frequency distribution plot for *Camelina sativa* cv Suneson using whole genome shotgun reads.

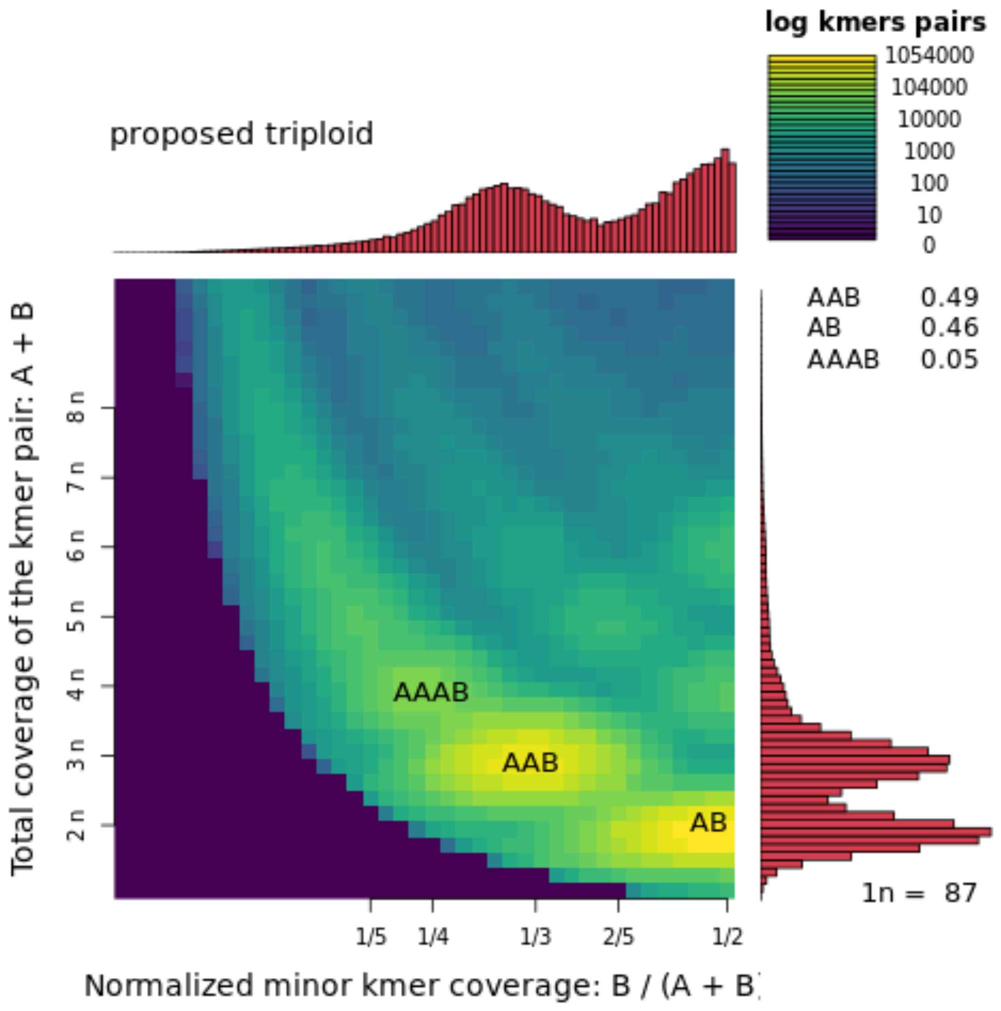

Supplementary Figure 2. Smudgeplot analysis of *Camelina sativa* cv. Suneson whole genome shotgun reads.

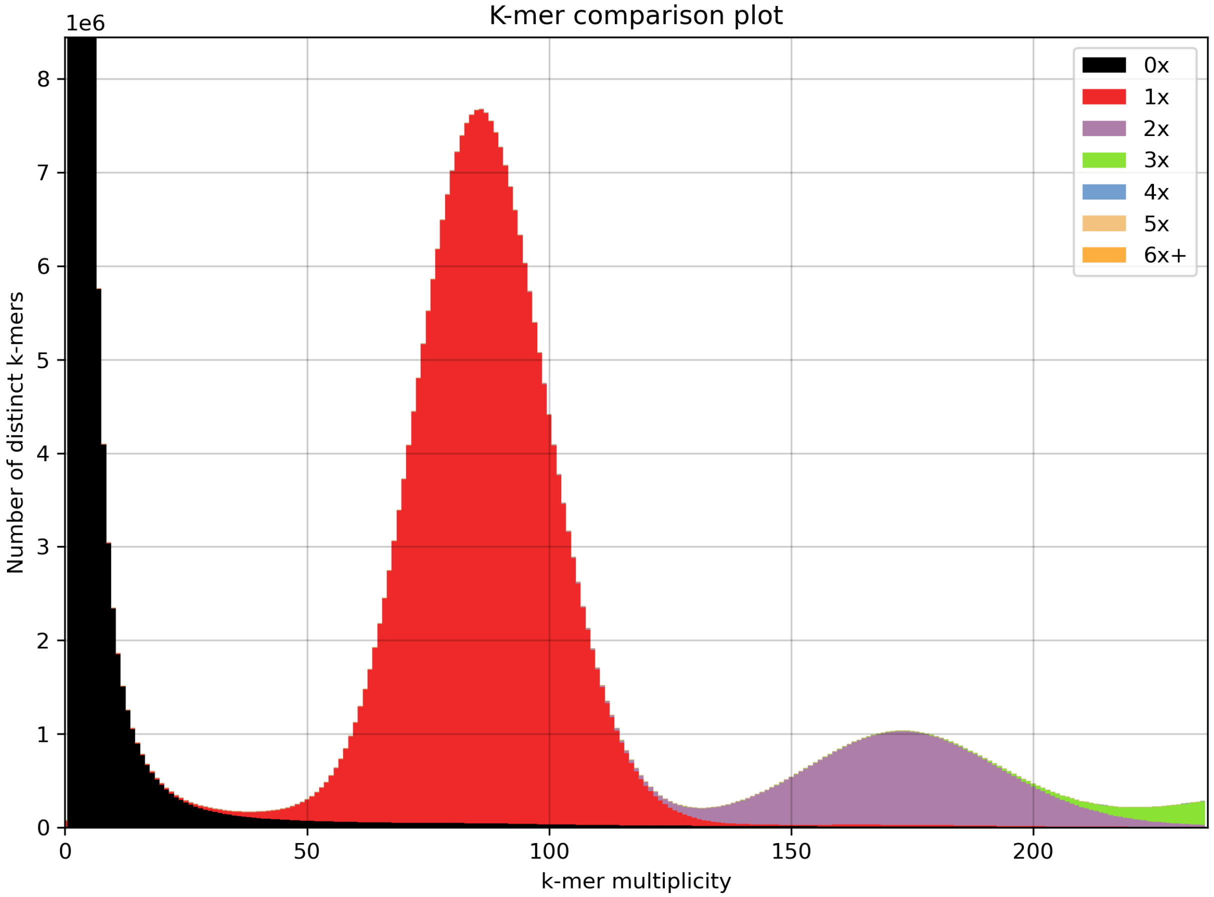

Supplementary Figure 3. KAT k-mer comparison plot of the *Camelina sativa* cv Suneson genome assembly.
